# Neuropsychological alterations in mercury intoxication persist several years after exposure

**DOI:** 10.1590/S1980-57642009DN20200003

**Published:** 2008

**Authors:** Elaine Cristina Zachi, Anita Taub, Marcília de Araújo Medrado Faria, Dora Fix Ventura

**Affiliations:** 1 M.Sc. Departamento de Psicologia Experimental, Núcleo de Neurociências e Comportamento, Instituto de Psicologia, Universidade de São Paulo, São Paulo SP, Brazil.; 2 M.Sc. Instituto de Psiquiatria da Universidade de São Paulo, São Paulo SP, Brazil.; 3 Ph.D. Departamento de Medicina Legal, Ética Médica e Medicina Social e do Trabalho, Faculdade de Medicina, Universidade de São Paulo, São Paulo SP, Brazil.; 4 Ph.D. Departamento de Psicologia Experimental, Núcleo de Neurociências e Comportamento, Instituto de Psicologia, Universidade de São Paulo, São Paulo SP, Brazil.

**Keywords:** mercury, occupational exposure, cognition disorders, neuropsychological tests, mood

## Abstract

Elemental mercury is a liquid toxic metal widely used in industry. Occupational exposure occurs mainly via inhalation. Previously, neuropsychological assessment detected deficits in former workers of a fluorescent lamp plant who had been exposed to elemental mercury vapor and were away from exposure for several years at the time of examination.

**Objectives:**

The purpose of this work was to reexamine these functions after 18 months in order to evaluate their progression.

**Methods:**

Thirteen participants completed tests of attention, inhibitory control, verbal/visual memory, psychomotor speed, verbal fluency, visuomotor ability, executive function, semantic knowledge, and depression and anxiety inventories on 2 separate occasions.

**Results:**

At baseline, the former workers indicated slower psychomotor and information processing speed, verbal spontaneous recall memory impairment, and increased depression and anxiety symptoms compared to controls (P<0.05). Paired comparisons of neuropsychological functioning within the exposed group at baseline and 1.5 years later showed poorer immediate memory performance (P<0.05). There were no differences on other measures.

**Conclusions:**

Although the literature show signs of recovery of functions, the neuropsychological effects related to mercury exposure are found to persist for many years.

## Abstract (pt)

Mercúrio metálico é um metal líquido e tóxico com ampla aplicação industrial. A exposição ocupacional ao metal ocorre principalmente por inalação. Previamente, comprometimento em várias funções neuropsicológicas foi constatado em ex-trabalhadores de fábrica de lâmpadas fluorescentes que haviam sido expostos ao vapor de mercúrio e estavam afastados da fonte de exposição por vários anos no momento do exame.

**Objetivos:**

Reexaminar estas funções após 18 meses, com a finalidade de verificar a possível progressão do comprometimento.

**Métodos:**

Treze participantes foram submetidos a testes de atenção, controle inibitório, memória verbal e visual, funções executivas, conhecimento semântico e inventários de depressão e ansiedade.

**Resultados:**

Na linha de base, os ex-trabalhadores mostraram lentificação psicomotora e no processamento de informações, déficit de memória verbal espontânea, além de maiores sintomas de depressão e ansiedade comparados com controles (P<0,05). Comparações pareadas do grupo exposto na linha de base e 18 meses após indicaram pior desempenho em memória imediata (P<0,05). Não houve diferença nas demais medidas.

**Conclusões:**

Embora a literatura mostre indícios de recuperação das funções, os efeitos neuropsicológicos relacionados ao mercúrio podem persistir por muitos anos.

Elemental mercury (Hg^0^) is a volatile, liquid metal at ambient temperature and its vapor is highly toxic. Despite its hazardous effects, elemental mercury is used industrially for the manufacture of a host of products, such as fluorescent lamps, thermometers, barometers, amalgams. Chronic occupational mercurialism is a set of symptoms which develop after a period of inhalation of elemental (Hg^0^) or inorganic mercury (Hg^2+^, Hg^3+^) vapor during work. Symptoms include motor impairment, salivation, insomnia, memory loss, gengivitis and erethism.^1,2^ The latter includes changes of personality and behavior such as irritability, agitation, mood lability, excessive shyness, and depression.^1,3-5^ Symptoms of mercurialism can be found in individuals with a history of chronic exposure to mercury vapor years away from the source of exposure.^1,2,5^

Long-term neuropsychological disturbance effects after the cessation of exposure to mercury vapor have received little research attention. A few case-control investigations have focused on this issue.^3-9^ Some of these studies did not find significant differences between exposed and controls in any of the cognitive functions assessed^7,9^ while others have suggested that deficits of verbal^5,8^ or visual memory,^6^ short term memory,^3,4^ attention,^6^ coordination,^3,4,8^ information processing speed,^5^ reaction time,^3,4^ and visuomotor function^6^ are likely to be persistent even years after chronic exposure. It is therefore reasonable to consider that the likelihood of reversibility both controversial and unclear.

This paper investigated the possibility of progressive neuropsychological dysfunction in former workers with history of mercury vapor exposure after a period away from the exposure source. In the present study, we compared two neuropsychological evaluations performed 18 months apart.

## Methods

The participants were thirteen former workers from a fluorescent lamp plant who had been diagnosed with mercurialism and received care at the Hospital das Clínicas (HC), Faculdade de Medicina da Universidade de São Paulo (FMUSP). The inclusion criteria were: chronic occupational mercurialism diagnosis due to past exposure to mercury vapor and no history of alcoholism, drug abuse, cerebrovascular or endocrine disease, head injury, or chelation treatment. Urinary mercury analyses were carried out by cold vapor atomic absorption spectrophotometry (details have been described previously).^5^

Each participant underwent a neuropsychological evaluation on two occasions, with a period of 18 months between the assessments. The neuropsychological battery included tests of attention (WMS Digit Span),^10^ inhibitory control (Stroop Interference Test),^11^ verbal memory (Buschke Selective Reminding Test - SRT),^11^ visual memory (WMS Visual Reproduction),^10^ verbal fluency (FAS),^11^ motor function (Grooved Pegboard),^12^ visual-spatial function (WAIS Block Design),^13^ executive function (Wisconsin Card Sorting Test),^14^ and semantic knowledge (WAIS Vocabulary).^13^ The Beck Depression Inventory – BDI^15^ and the State-Trait Anxiety Inventory – STAI^16^ were included in order to verify if potential changes in cognitive and motor functions were due to mood alterations.

These subjects had been assessed in a previous study in which 26 former workers participated.^5^ The previous sample’s results indicated slower information processing speed (Stroop Interference Test part 1 and 2), inferior performance in psychomotor speed (Grooved Pegboard), and in verbal spontaneous recall memory (SRT) (P<0.05) compared to 20 control subjects. In addition, the former workers showed higher depression and anxiety scores (P<0.001). The measures on Digit Span, Visual Reproduction, FAS, Block Design, Wisconsin Card Sorting Test, and Vocabulary were not statistically different between the 2 groups.

The present study was approved by the Ethics Committee of the Instituto de Psicologia (0403/CEPH/190803/IP), Universidade de São Paulo, São Paulo, Brazil, and all subjects gave written informed consent prior to testing. Demographic characteristics, medical data, and mercury exposure variables of the 13 former workers are summarized in Table 1.

**Table 1:** Demographic and medical characteristics and exposure to mercury-related variables, of the former workers.

	N	%	Mean±SD	Range
Sex				
Male	11	85		
Female	2	15		
Age (years)			42.3±5.2	35-56
Education (years)			9.1±1.7	8.5-11
Duration of exposure (years)			7.4±2.3	4-12
Period since removal from exposure (years)			7.7±3.2	4-12
U-Hg during the period of exposure (µg/g Cr)*			32.7±26.1	9-66
U-Hg 1 year after cessation of exposure (µg/g Cr)			1.8±1.4	0.8±1.8
Tendinitis diagnosis	6	46		
Psychoactive medication	1	8		
Antidepressants (tricyclics or serotonin reuptake inhibitors)	1	8		
Tranquilizers (benzodiazepines)	5	38		
Combined	6	46		
None				

Data are number of subjects (N), percentages (%) or means±standard deviations (SD), urine mercury concentrations (U-Hg), creatinine (Cr).

* Data from 7 participants. Brazilian limits of mercury in urine for workers and the general population are 35 µg/g Cr and 5 µg/g Cr, respectively.^1^

### Statistical analyses

Continuous data with non-normal distribution were log-transformed. T-tests for paired observations were applied to scores to assess differences among subjects between the baseline and second assessment. In order to verify interaction between cognitive scores from the last evaluation and predictor factors/covariates, the General Linear Model (GLM) was performed. Dependent variables were the neuropsychological measures. Age, raw score in the vocabulary test as a measure of pre-morbid general ability, depression score, and mercury-related variables (duration of exposure, period since removal from exposure, and urinary mercury concentration/U-Hg) were included as factors/covariates. Since anxiety showed a high correlation with depression, STAI scores were not included in the model. A P value <0.05was taken as indicative of significance.

## Results

Table 2 presents test results obtained both in the first assessment and the present study, along with the statistical difference between the two examinations. The Paired-Sample t tests revealed that the exposed group showed less perseverative errors on the Wisconsin test at the second evaluation, indicating executive function improvement. However, Digit Span Forward scores were significantly lower 1.5 years after the first assessment.

**Table 2:** Comparisons of cognitive score changes for the former workers.

	1st assessment Mean±SD (Md)	2nd assessment Mean±SD (Md)	P-value
Digit Span			
Forward	5.1±1.8 (5)	4.4±1.4 (3)	0.04*
Backward	4.1±1.3 (4)	3.8±1.5 (4)	0.51
Stroop^†^			
Part 1^‡^	18.1±6.6 (16.5)	20.6±13.8 (15)	0.10
Part 2^‡^	24.4±9.8 (22)	22.7±10.6 (19)	0.23
Part 3	34.1±13.5 (32.5)	40.4±24.1 (31)	0.81
SRT			
Total number of words	93.8±21.7 (86)	93.5±26.5 (100)	0.92
Long term recall^‡^	74.4±30.5 (62)	76.1±35.5 (90)	0.75
Consistent long term recall^‡^	46.8±28.8 (33)	45.3±33.1 (45)	0.78
Delayed recall^‡^	7.2±3.6 (7.5)	6.8±3.7 (6)	0.53
Recognition	10.5±2.1 (10.5)	9.0±4.5 (12)	0.62
Visual reproduction			
Immediate recall	24.2±8.7 (31.5)	25.1±4.9 (27)	0.66
Delayed recall	19.0±10 (20)	19.6±9.1 (20)	0.73
FAS	26.2±10.7 (26)	25.2±11.6 (23)	0.57
Grooved Pegboard^†^			
Dominant hand^‡^	83.7±12.3 (84)	80.4±18.2 (77)	0.63
Non-dominant hand^‡^	97.0±16.7 (93)	92.2±30.5 (86.5)	0.19
Block design	19.8±9.6 (20.5)	21.1±10.7 (25)	0.48
Wisconsin			
Errors	62.7±24.1 (66)	57.1±21.8 (64)	0.36
Perseverative errors	42.9±27.7 (33.5)	32.4±24 (27)	0.03*
Vocabulary	31.4±6.5 (34)	26.7±7.1 (24)	0.31
BDI^‡^	25.0±16.3 (24)	25.6±13.3 (22)	0.78
STAI			
State^‡^	51.7±10.7 (58.5)	52.8±9.3 (51.5)	0.38
Trait^‡^	54.4±15.4 (55)	55.6±12.2 (56)	0.88

SD: standard deviation; Md: median;

* P<0.05;

† Time in seconds was recorded;

‡ Measures on which former workers differed (P<0.05) from controls in the previous study (Zachi et al., 2007).^5^

It is important to note that previous analyses performed to evaluate the former worker’s neuropsychological results compared to controls, revealed several neuropsychological deficits (information processing speed, spontaneous recall verbal memory, and psychomotor speed) besides increased symptoms of depression and anxiety.^5^ However, scores concerning these measures did not differ between the first and the second assessments. Thus, the present longitudinal analysis provided no evidence of neuropsychological improvement.

The possibility of association between cognitive scores and predictor factors/covariates was investigated. In relation to exposure to mercury covariates, poorer performances on FAS (P=0.003) and on the Stroop test in part 3 (P=0.04) were explained by higher duration of exposure (not tabulated). Indeed, better scores on FAS were associated with a longer period since removal from exposure (P=0.002) (not tabulated). U-Hg was not associated with any neuropsychological measure. Age (P=0.03) and depression score (P=0.03) were also predictors for performance on Stroop 3 (not tabulated).

## Discussion

Investigations of occupational mercury-exposed groups after cessation of exposure have shown controversial results. Bast-Pettersen et al. (2005)^9^ did not find significant differences between former chloralkali workers and controls on any neuropsychological measure that was carried out (13.1 years of exposure on average, 5 years since the end of exposure). The authors suggested that long-term effects on neurobehavioral functions are more likely upon exposure to average levels of U-Hg between 30 and 40 nmol/mmol or higher. Letz et al. (2000)^7^ also reported no significant differences between former workers exposed to mercury for at least 4 months and a control group on neurobehavioral test results, 30 years after the exposure ceased.

On the other hand, previous studies have indicated cognitive and motor impairment in former plant workers with history of exposure to mercury vapor. Frumkin et al. (2001)^8^ found that former workers from a chloralkali plant, who were tested 5.73 years on average after the end of occupational exposure to mercury, performed worse than non-exposed individuals on tests of motor speed, coordination and verbal memory. Mathiesen et al. (1999)^6^ reported slow psychomotor speed, and visuomotor, visual memory and attention deficits among former chloralkali workers 12.7 years (average) after the exposure ceased. The disparity among results from previous studies may reflect different conditions concerning metal levels in air and the duration of exposure, which consequently are related to the amounts of mercury that crossed the blood-brain barrier, as well as urine, hair, and blood metal concentrations.

The results of the present analysis confirm previous data^5^ indicating that individuals with history of chronic exposure to mercury vapor retain cognitive deficits and increased mood symptoms years after the exposure period has ceased, since the scores found in the present assessment did not differ from those that had indicated cognitive impairment at baseline. The significant poorer scores on the Digit Span Forward at the second assessment are consistent with a previous study by Kishi et al. (1993,1994),^3,4^ which assessed ex-miners vs controls, 18 years after the closure of a mercury mine, suggesting that verbal short term memory seems to be affected even many years after exposure.

High BDI and STAI scores in both previous and the present studies suggest no alterations for mood since the first assessment. In fact, depression and anxiety are known symptoms of chronic mercurialism.^1,2^ However, the possibility that depression and anxiety could represent a reaction to their psychosocial context must be considered, which includes other medical conditions related to mercury exposure and unemployment.

We found association between duration of exposure and few words recovered on the FAS and worse performance on the Stroop test part 3, a measure of inhibitory control. Indeed, individuals with a longer period since removal from exposure were less likely to show a poor performance on the FAS. It seems that exposure to mercury vapor is associated with executive function effects that reduce after cessation exposure and with time. No association between U-Hg concentrations and neuropsychological performance was detected. This is explained by the fact that the kidneys eliminate large volumes of mercury only during and shortly after the period of exposure, consequently symptoms of mercury intoxication can be found in individuals with normal U-Hg.^1,2^

These former workers were part of a larger group in which visual functions were examined behaviorally and electrophysiologically. Statistical differences (p<0.05) were found between exposed subjects and controls for visual contrast sensitivity,^17,18^ visual field^19^ and color vision^17,20^ losses. Multi-focal and full field electroretinograms showed that these losses were in part caused by retinal impairment,^21^ but central nervous system components might have also contributed.

The limitations of this study that should be taken into account include the small sample size and the fact that the controls assessed in the first study (Zachi et al., 2007)^5^ were not reexamined. Indeed, the short period between examinations (1.5 years) could mask possible reversible effects.

In summary, the results of this study support the conclusion that cognitive and motor dysfunction in individuals with history of exposure to mercury vapor persist for several years. Longitudinal studies with many repeated measures are needed in order to verify the hypothesis that a given period is necessary to remedy neuropsychological impairment related to mercury exposure.
